# Intervention to enhance adherence to mandibular advancement appliance in patients with obstructive sleep apnoea: study protocol for a randomised clinical trial

**DOI:** 10.1186/s13063-021-05582-1

**Published:** 2021-10-13

**Authors:** Harishri Tallamraju, J. Tim Newton, Padhraig S. Fleming, Ama Johal

**Affiliations:** 1 Centre of Oral Bioengineering, Institute of Dentistry, Queen Mary University of London, Turner Street, London, E1 2AD UK; 2 Department of Population and Patient Health, Faculty of Dentistry, Oral & Craniofacial Sciences, King’s College London, London, UK

**Keywords:** Obstructive sleep apnoea, Mandibular advancement appliance, Patient adherence, Health Action Process Approach, Staged-matched intervention

## Abstract

**Background:**

Obstructive sleep apnoea (OSA) is a sleep-related breathing disorder characterised by the repeated episodic collapse of the upper airway during sleep, resulting in sleep deprivation, giving rise to apnoeas and hypopnoeas. Based on the severity of OSA, there are two primary treatment modalities, continuous positive airway pressure (CPAP) and mandibular advancement appliances (MAA); both are adherence-dependent. MAA is offered to those with mild to moderate OSA and is prescribed as an alternative to patients intolerable to CPAP. However, adherence to MAA treatment is variable and declines over time. Hence, the current study aims to assess the effectiveness of the stage-matched intervention, the Health Action Process Approach (HAPA), on adherence to MAA in patients with OSA.

**Methods:**

A single-centre randomised clinical trial will be undertaken at Bart’s Health NHS Trust. Fifty-six participants with newly diagnosed OSA are planned to be enrolled in the study and randomised to intervention care (IC) and standardised care (SC) groups. Participants in the SC group will receive routine care whilst participants in the IC group will receive the stage-matched intervention, developed using the HAPA model. Data indicating MAA adherence will be collected both objectively and subjectively, from micro-sensors embedded in the MAA design and sleep diaries, respectively at 3, 6, 18 and 36 months. In addition, a range of questionnaires designed to assess risk perception, outcome expectancy, and self-efficacy (SEMSA) and quality of sleep (PSQI and ESS) and life (EQ-5DL), socio-economic and social support scales will be used.

**Discussion:**

The currently available treatments for obstructive sleep apnoea depend entirely on the patient’s acceptance and use. There are several factors that affect cooperation and wear for example patients’ awareness of their condition, social support and psychological behaviour. In addition, mood, such as anxiety, stress, and depression, may affect wear. At the same time, we know that interventions involving more education and behaviour approaches can help patients adapt more easily to some treatments. As a result, the present trial aims to explore the potential role of these factors to maximise treatment success and minimise side effects.

**Trial registration:**

ClinicalTrials.gov NCT04092660. Registered on September 6, 2019

**Supplementary Information:**

The online version contains supplementary material available at 10.1186/s13063-021-05582-1.

## Administrative information

The order of the items has been modified to group similar items (see http://www.equator-network.org/reporting-guidelines/spirit-2013-statement-defining-standard-protocol-items-for-clinical-trials/).

**Table:** 

Title {1}	Intervention to enhance adherence to mandibular advancement appliance in patients with obstructive sleep apnoea: study protocol for a randomised clinical trial (IPOSAT)
Trial registration {2a and 2b}.	Clinicaltrials.gov ID NCT04092660 (9/13/2019)
Protocol version {3}	Version 04, 31.01.2020
Funding {4}	Self-funded
Author details {5a}	Harishri Tallamraju (HT): Research student Prof J Tim Newton (JTN): Research supervisor Prof Padhraig S. Fleming (PSF): Research supervisor Prof Ama Johal (AJ): Chief Investigator and Research supervisor
Name and contact information for the trial sponsor {5b}	Queen Mary, University of London Contact person: Dr. Mays Jawad Research & Development Governance Operations Manager Joint Research Management Office 5 Walden Street London E1 2EF Phone: 020 7882 7275/6574 Email: research.governance@qmul.ac.uk
Role of sponsor {5c}	Data management Record retention and Archiving Monitoring and Auditing Insurance and Indemnity

## Introduction

### Background and rationale {6a}

Obstructive sleep apnoea (OSA) is a sleep-related breathing disorder characterised by the repeated episodic collapse of the upper airway during sleep, resulting in sleep deprivation, giving rise to apnoeas and hypopnoeas [1].

OSA is the third most common respiratory disorder in the UK, affecting up to 10% of middle-aged adults [2, 3]. Severe long-term effects of this disease include excessive daytime sleepiness, cognitive dysfunction, hypertension, impaired quality of life, and increased cardiovascular morbidity and mortality [4].

An overnight sleep study is required to establish a diagnosis of OSA and provides the apnoea-hypopnoea index (AHI), which is the sum of the average number of apnoea (complete airflow cessation) and hypopnoea (partial airflow cessation) events per hour of sleep. The AHI has been classified into the following severity grades: mild (5–15), moderate (16–30) and severe (> 30) [5].

Based on the severity of OSA, there are two primary treatment modalities- continuous positive airway pressure (CPAP) and Mandibular advancement appliance (MAA) [6, 7]. CPAP is prescribed to those suffering from moderate to severe OSA. MAA is offered to those with mild to moderate OSA and is the alternative option for patients who cannot tolerate CPAP therapy [8]. It was noted that MAA reduces daytime sleepiness and improves the AHI by protruding the mandible and thereby maintaining an open pharyngeal airway [9]. However, studies have consistently demonstrated that CPAP is more effective than MAA at reducing sleep-disordered breathing and achieving complete control of OSA (AHI < 5) [10]. Despite the greater effect of CPAP on objective polysomnographic parameter (AHI), it does not appear to be more effective at achieving better health outcomes. It seems that the higher efficacy of CPAP is offset by greater MAA adherence. Phillips et al. (2013) showed that CPAP and MAA achieved similar improvements in excessive daytime sleepiness and quality of life. Average MAA adherence was 6.5 h/night compared to 5.2 for CPAP (*p* < 0.0001). These findings are consistent with the results of a recent systematic review and meta-analysis [11]. The meta-analysis presented that adherence was significantly lower with CPAP than MAA by 1.1 h per night (*p* = 0.004).

Although initial subjective treatment adherence to MAA therapy is relatively high, it is declining over time. A phone-based survey of 69 patients with mild to moderate OSA prescribed MAA, reported a 32% adherence rate after 4 years of therapy, indicating a high level of non-adherence [12]. The study also suggested the prevention of barriers, associated with MAA therapy adherence, to improve the efficiency of the appliance and disease outcome. In a systematic review, Hoffstein et al. (2007) reported a wide range of (4–76%) adherence rates in the first year of appliance use and further studies have highlighted that adherence decreases with time [13]: 83% after 1 year [14] and 62–64% after 4–6 year [15, 16].

MAA adherence might differ depending on the type of the appliance, custom-made or ready-made, disease severity, and perhaps patient management [17]. In a recent systematic review, patient-reported adherence (6.4–7 nights per week and 5–6.3 h per night) and preference (*p* ≤ 0.001) were strongly associated to custom-made MAA in comparison to ready-made MAA [18]. Subjective side effects such as dry mouth, excessive salivation, tooth discomfort, muscle tenderness, and jaw stiffness, have never lead to treatment discontinuation [19–22]. In addition, tooth movement and occlusal changes have been seen in objective measurements after 1 to 4 years of follow-up, but these changes have not been reported as being related to treatment withdrawal [23–27].

Dieltjens et al. (2013) were the first to investigate the impact of type D personality disorder on adherence to MAA therapy. Individuals with Type-D personality often possess a negative outlook towards life and are overwhelmed with emotions such as stress, anxiety, and anger. The study assessed 113 patients using two questionnaires: type D scale 14 and a postal questionnaire addressing side effects and adherence to MAA. The study included 83 patients with a baseline type D personality and reported a 45% non-adherence rate amongst them [14]. Patients with a type D personality had a higher discontinuation rate and lower adherence. These findings are in agreement with similar observations reported by Brostrom et al. (2007) in regards to lower CPAP adherence with type D personality [28]. Nevertheless, the study has some limitations. The first limitation of the study is the relatively small sample size (sample bias) and the non-meaningful comparison groups, which may reduce the generalisability of the findings. The second limitation is the subjective assessment of adherence and perceived side-effects rather than employing objective means of adherence measurements. A further limitation is that the characteristic negative reporting trait of the type D patients may cause them to overestimate their non-adherence [14].

Research shows that adoption of a new health behaviour, like a new physical activity routine or adhering to a prescribed medication regimen, is a challenging endeavour involving a variety of social, emotional, and cognitive factors [29]. A multicentre study examined perceived effectiveness, self-efficacy and social support among 122 adult patients with OSA aged ≥ 65 years prescribed for MAA therapy [30]. With a 30% response rate (*n* = 39), the study reported low rates of perceived effectiveness, self-efficacy and social support highlighting the lack of self-efficacy, expectations for positive outcomes and social support experienced by the older patients in the sample. These findings are debatable as literature has identified psychological and social factors and cognitive perceptions as determinants of CPAP adherence, including patients’ risk perceptions, treatment outcome expectations, locus of control, and self-efficacy [31–33]. Another possible limitation of the study is that the questionnaires were mailed to the participants, who were only contacted once by the authors explaining the poor response rate. As a result, further research involving larger samples of men and women incorporating modes, which increase the response rate, is necessary to gain a better understanding of the patient's perception of MAA therapy. In turn, this understanding could be implemented for the development of interventions enhancing patient's adherence and experience to MAA treatment.

Interestingly, previous studies in the field of Sleep Medicine have featured an aspect of enhanced patient education ranging from telephone support to home visits, motivational enhancement, or augmented support [34, 35], which have been proven to effectively improve CPAP adherence when compared to usual care. Bakker et al. (2016) conducted a randomised controlled trial of CPAP use, with motivational enhancement (ME) or CPAP only in 83 participants, with moderate to severe OSA. The trial demonstrated a clinically significant increase in CPAP adherence in the intervention arm (CPAP with ME), supporting the use of a motivational enhancement approach to optimise the management of OSA [36].

A recent Cochrane review has also emphasised the efficiency of interventions in enhancing adherence to CPAP by stating that educational, supportive and behavioural interventions increase CPAP usage to varying degrees [37]. Stage theories such as the Health Action Process Approach (HAPA), a social cognition model for behaviour change, can be used to identify factors to target in an intervention and their interrelationships. The model-based approach often uses a quantitative, questionnaire-based approach to assess a small set of factors linked within a model specifying how the factors are related to behaviour and to one another. It includes self-efficacy, outcome expectancies, and risk-perception as distal predictors, intention as a middle-level mediator, and volitional factors (such as action planning) as the most proximal predictors of behaviour. All are considered as determinants of adherence in CPAP therapy [38, 39].

A number of randomised controlled trials within medicine have examined the concept of stage-matched interventions based on HAPA, for example in the context of dietary behaviours [40], physical activity [41] and dental hygiene [42]. In order to investigate the efficiency of interventions on CPAP adherence, 110 OSA (AHI ≥ 15 events/h) patients were randomly assigned into staged-match intervention care (SMC) and standardised care (SC) groups [4]. The staged-match intervention care design, following the principles of HAPA, significantly improved CPAP adherence whilst facilitating intention formation and enhancing treatment self-efficacy. Although the evidence for stage theories is somewhat inconsistent, a meta-analysis [43] suggests that tailoring interventions to behavioural stages is more effective than a generic, non-staged-tailored approach.

The above stage theory advocates that behaviour intervention takes account of the stages of change. Individuals are presumed to progress through an ordered set of stages whilst contemplating, initiating, and maintaining health behaviour change [44]. Risk perception is an antecedent, that forms an intention to adopt a precautionary action or treatment [4]. After a treatment intention develops, it transforms into detailed action plans and these plans may promote moving further into action and/or a maintenance stage [45] implies that understanding behaviour change over time, on dynamic variables instead of static variables, would achieve maximum intervention effectiveness [4]. This feature differentiates stage theories from social cognition theories, such as the theory of planned behaviour (TPB), which interpret behaviour change as a continuous process.

The approach also recommends a distinction between pre-intentional motivational processes and post-intentional volitional processes [42]. The motivational phase comprises of growing risk perception and outcome expectancies, leading to the development of an intention. Although risk perception is the initial step for developing an intention, it alone is deficient and outcome expectancies, characterised by the advantages and disadvantages of the health behaviour in context are essential to promote intention formation. For example, the more a patient feels vulnerable to the possible health threats of long-term untreated OSA (hypertension, cardiovascular diseases), the more he or she will expect from the MAA therapy.

In the volitional phase, Intention and behaviour implementation (adherence to MAA) would be mediated through action planning [46]. Action planning consists of specifying when, where and how to perform the behaviour [47]. Self-efficacy is an important construct to consider for behaviour change [48] and facilitates maintenance of action [46]. For example, after a patient is provided MAA treatment, he or she would make concrete plans concerning the commencement and continuation of the treatment whilst overcoming the difficulties. Highly self-efficacious individuals are more confident in coping with setbacks and easily tackle unanticipated difficulties, as opposed to individuals with low self-efficacy (Bandura, 1997). Since patients with OSA experience different mindsets from initiation to long-term adherence, it is imperative to frame interventions to optimise adherence, tailor-made to the patients’ specific psychological variables at different stages of therapy [4].

In summary, research on increasing adherence to mandibular advancement appliances in obstructive sleep apnoea patients in underrepresented, which is in sharp contrast to the literature regarding CPAP adherence. Thus, we aim to address this shortfall by identifying the factors influencing adherence to MAA in patients with OSA and simultaneously assess the effectiveness of stage theories/stage-matched intervention on subjective and objective adherence, using the HAPA model.

### Objectives {7}

#### Primary objective

##### Aims and research questions

The aim and primary outcome of this study is as follows:

- To assess the effectiveness of stage-matched intervention on adherence to mandibular advancement appliances (MAA) in patients with obstructive sleep apnoea (OSA).

##### Secondary objectives

The secondary outcomes are as follows:

- To identify the psychosocial and socio-economic indicators enhancing adherence of MAA in patients with OSA.
- Use of the indicators to develop a psychological and socio-economic predictor model.

##### Null hypothesis

Stage-matched intervention does not enhance adherence to MAA in patients with OSA compared to standardised care.

#### Primary endpoint

Adherence i.e. the number of the hours the patient uses the appliance every night will be measured both objectively and subjectively at 3, 6, 18 and 36 months to assess the effectiveness of the interventions in enhancing adherence to MAA.

#### Secondary endpoint

Self-efficacy, risk perception, outcome expectancy, socio-economic status and social support will be measured to assess the ability of these variables in predicting adherence.

### Trial design {8}

This study is a single-centre, superiority, two-arm, parallel-group, individually randomised clinical trial, with 1:1 allocation, designed to test the effectiveness of stage-matched intervention in enhancing patient adherence to MAA therapy in patients with OSA. In addition, we plan to recruit patients with a confirmed OSA diagnosis, specifically referred for MAA therapy from secondary care.

Ethical approval was obtained from the Greater Manchester West Research ethics committee (REC ref: 19/LO/0972 and 19/NW/0391). Patients meeting the eligibility criteria will be provided with a patient information leaflet explaining the whole study. Interested patients will be asked to sign the informed consent, after which they will be randomly assigned into two groups- intervention care (IC) and standardised care (SC). Only the investigator and chief investigator (CI) will be aware of the participant’s allocation.

Participants will be provided with a sleep diary (Additional file 1) to record their hours of sleep and MAA wear-time, which will give a subjective record of the adherence (duration of usage of MAA). The TheraMon®, a micro-sensor included in the MAA design, will be used for the objective documentation of MAA adherence. TheraMon® calculates the actual wear time by measuring temperature every 15 min and then transforms this information into wear time when the temperature ranges between two specific values. In the present study, this range is defined as 28 °C to 38 °C, which includes the vast majority of intraoral temperature values observed in an individual under normal conditions.

At baseline (T0), part of the routine clinical subject demographics to be collected will include age, gender, body mass index (BMI), and neck circumference. The AHI will also be recorded during the initial screening. Following an oral examination, upper and lower alginate impressions will be taken along with the participant's bite. In addition, participants will be asked to complete the following questionnaires at T (0):

- Epworth Sleepiness Scale (ESS)
- Self-efficacy measure for sleep apnoea (SEMSA) modified for oral appliance
- Pittsburgh Sleep Quality Index (PSQI)
- EuroQol-5 Dimension (EQ-5D)
- Socio-economic position questionnaire
- Social support scale

The above questionnaires will provide information concerning the participant’s daytime sleepiness, personality, quality of sleep, health-related quality of life, socio-economic status and social support, respectively.

At pre-treatment T(1), participants will undergo the initial fitting of the MAA and be provided instruction on use and care.

Both IC and SC groups will be called for follow-up at 3 (T2), 6 (T3), 18 (T4) and 36 (T5) months. Any problems that the patients might be experiencing regarding MAA use will be attended to at these appointments. In addition, data indicating adherence will be collected and evaluated at the appointments both subjectively and objectively by downloading the data from the sensor using dedicated software.

Furthermore, at T(2), participants will be asked to complete the SEMSA questionnaire, whilst at T(3), participants will need to complete the ESS, SEMSA, PSQI and EQ-5D questionnaires.

One-to-one interviews will be conducted 6 months into treatment with both (*n* = 5–10) compliant and non-compliant patients. It will comprise of questions, which will address the following topics:

- Patient's awareness of risks and benefits of OSA
- Barriers and facilitators of MAA therapy
- The interviews will be conducted face-to-face, recorded, and transcribed by a third-party service (Essential secretary Ltd., UK). However, due to the COVID-19 pandemic, the interviews will take place online (Microsoft® Teams or Zoom cloud meetings) to ensure the participant’s safety. Although, if the participant is comfortable travelling, the interview can be carried out in a private seminar room. Inductive content analysis will be employed to describe the experiences of OSA and MAA therapy of compliant and non-compliant patients without imposing preconceived categories and names of categories to evolve from the data [49].

## Methods: Participants, interventions and outcomes

### Study setting {9}

The study will be undertaken at the Royal London Dental Hospital, Bart’s Health NHS Trust in line with the CONSORT guidelines.

### Eligibility criteria {10}

#### Inclusion criteria

- Adult (≥ 40 years old)
- Confirmed diagnosis of OSA (AHI ≥ 5)
- Referred for MAA therapy
- Must be able to understand, read and write English; with the assistance of a translator

#### Exclusion criteria

- Insufficient teeth for MAA fabrication
- Poor dental or periodontal health
- Symptomatic temporomandibular disorder (TMD)
- Previously used an MAA
- Patients with uncontrolled epilepsy

### Who will take informed consent? {26a}

In line with standard care, patients will be provided information regarding the study via a Participant Information sheet and what will happen to them if they agree to participate. Once the patients have agreed to participate, the research student (HT) will take written informed consent from the participants.

### Additional consent provisions for collection and use of participant data and biological specimens {26b}

Not applicable

## Interventions

### Explanation for the choice of comparators {6b}

Participants in the SC group will receive the MAA and the routine care except for the staged-matched intervention.

### Intervention description {11a}

Participants in the IC group will receive additional support in the form of behaviour change intervention. The behaviour change intervention based on the HAPA model entails delivering interventions in a staged manner. The interventions that will be provided are described in Table 1, along with the time point (Table 2).

**Table 1:** Intervention care content specified by behaviour change techniques and linked to constructs of the HAPA model

Time Point	Intervention component	Behaviour change technique	HAPA construct targeted	Content
T(1)	Pamphlet	Information about consequences	Risk perception and outcome expectancy	The health pamphlet (Additional file 1), based on HAPA, divided into 6 sections, systematically provides information about the risk, benefits and treatment of OSA. Section 2 and 4 of the pamphlets provides spaces for the participants to discuss about risks and benefits that concern them the most. Section 3 explains the various treatments available and a brief explanation of the mechanism of the treatment.
		Action planning	Task self-efficacy	Sections 5 and 6 provide valuable information for participants on how to begin with using the appliance and to continue using it regularly. Patients are advised that is normal to struggle at time when starting a new habit and setting short-term attainable goals will aid them in their struggles to adjust. The section suggests participants to seek feedback from their family, especially their sleeping partner regarding their improvement. This type of support motivates the patient to work more towards achieving their goals.
		Goal setting		
		Problem-solving		
		Social support (unspecified)		
		Self-reward	Coping and recovery self-efficacy	In section 6, participants are told about the importance of rewarding themselves about the effort they put in to achieve their goals.
		Relapse prevention	Recovery self-efficacy	Section 6 also advises the participants to focus on the positives and think about situations that effect their capability and then about options to avoid/cope with these situations.
T(1)	Video	Credible source	Risk perception, outcome expectancy and task and coping self-efficacy	Patients will be shown a video [50] of an OSA patients who are undergoing treatment, in order for them to relate to someone who is going through the same condition as him/her. The video consists of patients talking about how OSA effected their life and its negative consequences. The patients will also talk about what motivated them to start the treatment, how has it changed their life and what does they do to use the appliance regularly. It will also consist of a specialist in the field of OSA, briefly talking about the ill effects of untreated OSA and the specific oral appliance treatment available.
		Social comparison		
		Information about the negative and positive consequences		
		Demonstration of behaviour		
		Feedback on the behaviour		
		Social support (emotional and practical)		
T(1)	Counselling	Information about health consequences	Risk perception, outcome expectancy and task self-efficacy	Participants will be given an initial counselling session in person along with their partner if they wish. During the session i.e. structured to fit the participants needs… • Their knowledge regarding OSA will be assessed • The above video will be shown • Using the information provided on the pamphlets the risks of untreated OSA and the benefits of the treatment will be discussed • If the partner is present at the appointment, they will be asked to complete a section of the pre- screening questionnaire which is part of the routine clinical examination. In the questionnaire, the partner will also be asked to indicate their and the participant’s quality of sleep. In addition, the partner will also be asked to indicate the severity of the participant’s snoring and whether it has an influence on their sleep.
		Social Support (unspecified and emotional)		
		Verbal persuasion about capability		
T(2), T(3), T(4) and T(5)	Follow up at sleep clinic	Monitoring the behaviour and the outcome	Coping and recovery self-efficacy	Participants would be required to visit the sleep clinic for follow up at months 3 and 6. Their MAA usage will be assessed both objectively and subjectively by downloading the data from the micro-sensor chip embedded in the appliance and by recording the hours from their daily sleep log respectively.
		Focus on past success and verbal persuasion about capability	Coping and recovery self-efficacy	Feedback would be provided depending on the participant’s usage. Their planning sheets would be discussed, and appropriate feedback will be provided whilst encouraging them to set more active goals and plans.
		Social comparison	Coping and recovery self-efficacy	To increase their emotional support other patient’s feedbacks and successful treatment would be shared.
		Problem-solving	Coping and recovery self-efficacy	Additionally, participants will be prompted to identify common factors that act as barriers for them in using the appliance and will be helped to find solutions to overcome such factors tailored to the participants needs.
3, 6, 18, and 21 weeks	Booster phone calls	Verbal persuasion about capability	Coping and recovery self-efficacy	Participants will receive calls at weeks 3, 6, 18 and 21 approximately 10–15 min in duration, prompting them to keep working towards their goals and stating that they are capable of achieving them.
		Social support unspecified	Coping and recovery self-efficacy	Participant’s partner’s experience of the treatment will also be discussed by asking them to share their thoughts on the participant’s improvement.
		Problem-solving	Coping and recovery self-efficacy	Additionally, participants will be prompted to identify common factors that act as barriers for them in using the appliance and will be helped to find solutions to overcome such factors tailored to the participants needs.

**Table 2:** Intervention care and standardised care components

Time point	Intervention care group	Standardised care group
*T0 (Baseline)*	• Questionnaires - ESS, SEMSA (Modified for Oral Appliance) PSQI, EQ-5D, Socio-Economic Position and Social Support	• Questionnaires - ESS, SEMSA, PSQI, EQ-5D, Socio-economic position, Social Support scale
*T1 (Pre-treatment)*	• Providing instruction about how to use the MAA • Health pamphlet based on HAPA theory • Assessment of knowledge of OSA and involvement of the partner in education • A brief education focusing on the benefits associated with MAA • 10-min video education about OSA, emphasising the negative consequences if OSA is untreated • Risk perception communication • Setting Goals and action planning (steps to facilitate the use of MAA)	• Providing instruction about how to use the MAA • Health pamphlet about OSA and MAA
*T2 (3 months into treatment)*	• Phone calls to be made at 3 and 6 weeks - Problem-solving - Evaluation of the patient and partner perception of treatment - Verbal encouragement - Setting attainable goals and action planning • Follow up at Sleep Clinic -Assessment of MAA use -Problem Solving -Verbal Encouragement -Sharing other Patient’s Feedbacks and successful treatment experiences • Questionnaire -SEMSA	• Follow up at Sleep Clinic - Problem-solving - Assessment of MAA use • Questionnaires -SEMSA
*T3 (6 months into treatment)*	• Phone calls to be made at 18 and 21 weeks - Problem-solving - Partner involvement in treatment and family support - Verbal encouragement - Setting attainable goals and action planning • Follow up at sleep clinic - Assessment of MAA use - Problem-solving - Verbal encouragement - Sharing other patient’sfeedbacks and successful treatment experiences • Questionnaires - ESS, SEMSA, PSQI, EQ-5D • One-to-one interviews	• Follow up at Sleep Clinic - Problem-solving - Assessment of MAA use • Questionnaires - ESS, SEMSA, PSQI, EQ-5D • One-to-one Interviews
*T4 (18 months into treatment)*	• Assessment of MAA use	• Assessment of MAA use
*T5 (36 months into treatment)*	• Assessment of MAA use	• Assessment of MAA use

### Criteria for discontinuing or modifying allocated interventions {11b}

As we are comparing the benefits of supportive information on participant’s use of a standard treatment undertaken by NHS, we do not see any specific criteria for discontinuing or modifying allocated interventions.

### Strategies to improve adherence to interventions {11c}

The study specifically aims to enhance MAA therapy adherence in patients with OSA. The strategies employed to improve adherence to the intervention i.e. the MAA therapy have been designed on the principles on the behaviour change model, HAPA. These strategies are described in detailed in Table 1.

### Relevant concomitant care permitted or prohibited during the trial {11d}

No relevant concomitant care is prohibited during the trial.

### Provisions for post-trial care {30}

Mandibular advancement therapy will continue for all patients post the trial.

### Outcomes {12}

#### Primary outcome

Adherence i.e. the number of hours the patient uses their appliance every night will be measured both objectively and subjectively at 3, 6, 18 and 36 months to assess the effectiveness of the interventions in enhancing adherence to MAA.

#### Secondary outcome

Self-efficacy, risk perception, outcome expectancy, socio-economic status and social support will be measured to assess the ability of these variables in predicting adherence

### Participant timeline {13}

The participant timeline is found in Table 3.

**Table 3:** Schedule of assessment

Assessment	Baseline T0	Pre-treatment T1	3 months T2	6 months T3	18 months T4	36 months T5
Questionnaires						
Epworth Sleepiness Scale (ESS)	X			X		
Self-Efficacy Measure for Sleep Apnea	X		X	X		
(SEMSA)				X		
Pittsburgh Sleep Quality Index (PSQI)	X			X		
EQ-5D	X					
Socio-Economic Questionnaire	X					
Social Support Questionnaire	X					
Age	X					
Gender	X					
Body mass index (BMI)	X					
Neck circumference	X					
Objective measure of adherence			X	X	X	X
Subjective measure of adherence			X	X	X	X
One-to-one interviews				X		

### Study scheme diagram

**Fig. 1:**
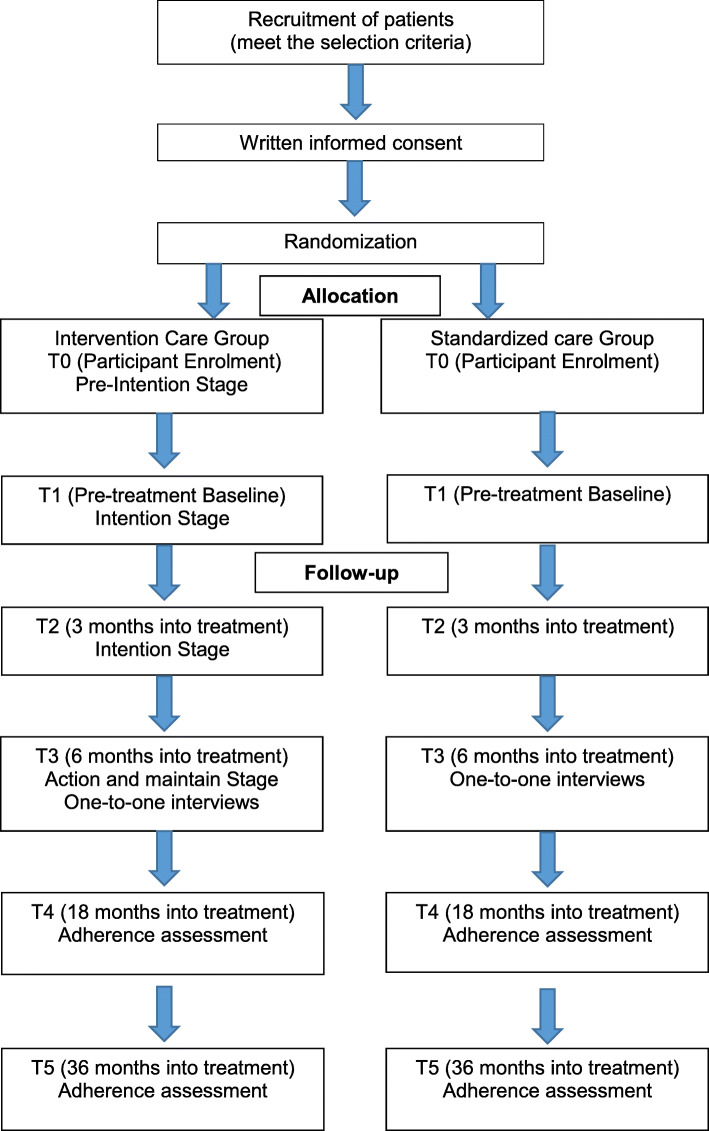
Study Flow diagram

The study scheme diagram is found in Fig. 1.

### Sample size {14}

A minimum sample size of 56 patients distributed in two groups is proposed for given α (0.01) and power (0.9), for 2 randomly assigned groups, intervention care (*n* = 28), standard care group (*n* = 28). Finally, assuming a maximum of 15% dropout, a total number of 64 patients is required.

### Recruitment {15}

As part of the routine care for patients presenting with sleep disordered breathing, they are all diagnosed with OSA, on the basis of an overnight sleep study, performed in sleep clinics. Patients requiring mandibular advancement appliance (MAA) therapy are then referred to specific dental sleep clinics for treatment, as part of a multi-disciplinary approach to care. We plan to recruit potential patients, with a confirmed diagnosis of OSA, that are specifically referred for MAA therapy to Barts’ Health NHS Trust.

## Assignment of interventions: allocation

### Sequence generation {16a}

A simple computer-generated randomisation method will be performed using a restricted (10-number block) random number sequence (www.graphpad.com/quickcalcs/randomn2.cfm) to ensure equivalence of numbers in each group. Participants will be stratified by OSA severity. In every 10-number block from the random table, the sequence will be checked to ensure the even numbers are equal to the odd numbers. Each number in the random table will be given a study number and assigned into one of the study groups.

### Concealment mechanism {16b}

A table for the allocation of the participants in the study will be composed and kept in a sealed opaque envelope by the CI.

All the documents related to the randomisation, and allocation sequence generation will be kept in a box in a locked cabinet away from the clinical environments in the CI’s office.

### Implementation {16c}

Research student (Harishri Tallamraju)

## Assignment of interventions: Blinding

### Who will be blinded {17a}

Only the research student (HT) and CI will have access to the Master file and patient allocation. Patients will be anonymised and allocated a unique study number and the data then blinded to the statistician, for analysis.

### Procedure for unblinding if needed {17b}

Not applicable

## Data collection and management

### Plans for assessment and collection of outcomes {18a}

- Questionnaire responses
- Mandibular advancement appliance with an in-built sensor will be used to collect objectively measured adherence

### Plans to promote participant retention and complete follow-up {18b}

Data concerning adherence will be retained.

### Data management {19}

The data recorded in this study will be stored securely both physically and electronically. The data will be accessed at the Queen Mary University of London by the CI and research student only and will be password protected. The personal data of the participants will be securely held within the research facilities at the Queen Mary University of London, by the chief investigator. Data transfer for analysis will be undertaken in an anonymised form, using unique ID numbers and password-protected access.

### Confidentiality {27}

Information related to participants will be kept confidential and managed in accordance with the Data Protection Act, NHS Caldecott Principles, The Research Governance Framework for Health and Social Care, and the conditions of Research Ethics Committee Approval.

### Plans for collection, laboratory evaluation and storage of biological specimens for genetic or molecular analysis in this trial/future use {33}

Not applicable

## Statistical methods

### Statistical methods for primary and secondary outcomes {20a}

Both objective and subjective data of adherence i.e. duration of the usage of the appliance will be collected at 3, 6, 18 and 36 months will be analysed to assess the effectiveness of Intervention care in comparison with standardised care.

Comparison of pre- and post-treatment scores of ESS, SEMSA, PSQI, and EQ-5D, for both the groups, will aid in identifying significant differences in terms of improvement.

Statistically, evaluate the ability of the variables -self-efficacy, risk perception, outcome expectancy, social support and socio-economic position, to predict patient adherence at 3, 6, 18 and 36 months.

#### List of statistical procedures

Assuring random assignment to groups, for this purpose, we need to test whether the observations differ in any of the time zero measurements. Indifference means there is no allocation bias.

T-test for each of the variables between SC and IC

1. Risk perception
2. Outcome expectancy
3. Self-efficacy
4. ESS – time zero
5. PSQI – time zero
6. EQ5D – time zero
7. Social support
8. Socio-economic (ordinal) – chi-square test

In case of violation of the *t* test assumption (normality of the error term), Wilcoxon tests will be implemented.

#### Assessing the aims of the study

In order to assess the aims of the study, we will test each of the repeated measures first by themselves using a repeated measures ANOVA controlling for groups:

1. Adherence – reported, times 3, 6, 18, 36 difference 3–6, 6–18, 18–36
2. Adherence – objective, times 3, 6, 18, 36 difference 3–6, 6–18, 18–36
3. ESS, time 6, difference 0–6
4. PSQI, time 6, difference 0–6
5. EQ5D, time 6, difference 0–6
6. Risk perception, times 3, 6, difference 0–3, 0–6, 3–6
7. Outcome expectancy, times 3, 6, difference 0–3, 0–6, 3–6
8. Self-efficacy, times 3, 6, difference 0–3, difference 0–6, 3–6

Once we understand the trends in the data, we can construct multiple regressions for Adherence variables. These can be the final measures from time 6 or the differences between them and previous times, 0 or 3. In these models, we will use the Psychosocial variables and socio-economic as predictors, beyond the group classification.

### Interim analyses {21b}

The Cl will make the final decision to terminate the trial.

### Methods for additional analyses (e.g. subgroup analyses) {20b}

Not applicable

### Methods in analysis to handle protocol non-adherence and any statistical methods to handle missing data {20c}

All randomised participants will be included in the main analyses. An intention-to-treat analysis will be carried out relating to any protocol non-adherence or dropouts. However, due to the nature of the trial very few dropouts are anticipated. Nevertheless, sensitivity analysis will be performed for any of the missing data.

### Plans to give access to the full protocol, participant level-data and statistical code {31c}

Not applicable

## Oversight and monitoring

### Composition of the coordinating centre and trial steering committee {5d}

Not applicable

### Composition of the data monitoring committee, its role and reporting structure {21a}

The data recorded in this study will be stored securely both physically and electronically. The data will be analyzed in the Queen Mary, University of London by the CI of the research student. The data will be password protected and the only the CI and the research student will have full access. The personal data of the participants will be securely held within the research facilities at the Queen Mary, University of London, by the chief investigator.

### Adverse event reporting and harms {22}

We expect no adverse events in the study, as it is questionnaire-based and relates to routine clinical care of MAA therapy in relation to OSA management.

### Frequency and plans for auditing trial conduct {23}

The sponsor or delegate retains the right to audit any study, study site or central facility. In addition, the funders may audit any part of the study where applicable.

### Plans for communicating important protocol amendments to relevant parties (e.g. trial participants, ethical committees) {25}

Any protocol amendments will be communicated to the sponsor (Joint Research Management Office), the relevant research ethics committee and to the study participants.

### Dissemination plans {31a}

It is intended that the results of the research would be presented at conferences or organising/attending workshops. In addition, the results will be published in a peer-review journal.

## Discussion

Not applicable

## Trial status

Protocol version 04, Dated: 31/01/2020

Date recruitment began: December 2019

Approximate date of recruitment completion: September 2021

## Supplementary Information

**Additional file 1.**

## Data Availability

Not applicable

## Abbreviations

AHI: Apnoea/Hypopnoea index
CI: Chief investigator
BMI: Body mass index
CPAP: Continuous positive airway pressure
ESS: Epworth Sleepiness Scale
EQ-5D: EuroQol-5 Dimension
HAPA: Health Action Process Approach
IC: Intervention care
MAA: Mandibular advancement appliance
OSA: Obstructive sleep apnoea
PSQI: Pittsburgh Sleep Quality Index
QUIPS: Quality in prognosis studies
SEMSA: Self-efficacy measure for sleep apnoea
SC: Standardised care
T0: Baseline
T1: Pre-treatment
T2: 3-month follow-up observation
T3: 6-month follow-up observation
T4: 18-month follow-up observation
T5: 36-month follow-up observation
