# Supplementary material for: Intervention to enhance adherence to mandibular advancement appliance in patients with obstructive sleep apnoea: study protocol for a randomised clinical trial
Source: Trials. 2021 Oct 13;22:699. doi: 10.1186/s13063-021-05582-1 (PMC8511865; doi:10.1186/s13063-021-05582-1)

Appendix 1


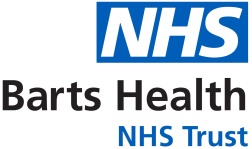


**DAILY SLEEP DIARY**

**(VERSION 2.0, 22.07.2019)**

Participant identification number for the study: __________

Title of the study: Intervention to enhance adherence to mandibular advancement appliance in Patients with Obstructive sleep apnoea: A randomised clinical trial

To help us understand your sleep problems, we need a report of the times when you sleep, nap and how often you wake during sleep. In addition, we need to know the times when you drink coffee, tea and alcoholic beverages.

If medication is taken, record the time medication is needed.

**IT IS IMPORTANT THAT YOU KEEP THIS RECORD FOR 7 DAYS.** Each column begins a new day; the first column is an example for you to study.

| Start Date | Example | Day 1 | Day 2 | Day 3 | Day 4 | Day 5 | Day 6 | Day 7 |
| --- | --- | --- | --- | --- | --- | --- | --- | --- |
| Naps: times & length you napped | 2:00 pm  45 min  6:30 pm  30 min |  |  |  |  |  |  |  |
| Medication: Amount & Time taken | Zimovane  5mg x 1  10:30 pm |  |  |  |  |  |  |  |
| Coffee & Tea  No. of cups/time | C. x 1  7:00 am  T. x 2  6:30 pm |  |  |  |  |  |  |  |
| Alcohol:  No. of units/time | A. x 2  7:00 pm  A. x 1  10:00 pm |  |  |  |  |  |  |  |
| Time in bed before lights out: | 30 min |  |  |  |  |  |  |  |
| Lights out: | 11:00 pm |  |  |  |  |  |  |  |
| Estimated time to fall asleep | 45 min |  |  |  |  |  |  |  |
| Estimated number of awakening in night & duration | 2:00 am  @20 min  4:30 am  @ 1 hour |  |  |  |  |  |  |  |
| Time of awakenings | 7:30 am |  |  |  |  |  |  |  |
| Total hours of sleep | @ 7hrs |  |  |  |  |  |  |  |
| Overall sleep quality night:  Poor= 1  Average= 2  Good= 3 | 3 |  |  |  |  |  |  |  |

If you have any queries please contact Prof. Ama Johal via his secretary at The Royal London Hospital on 0207 377 7379.


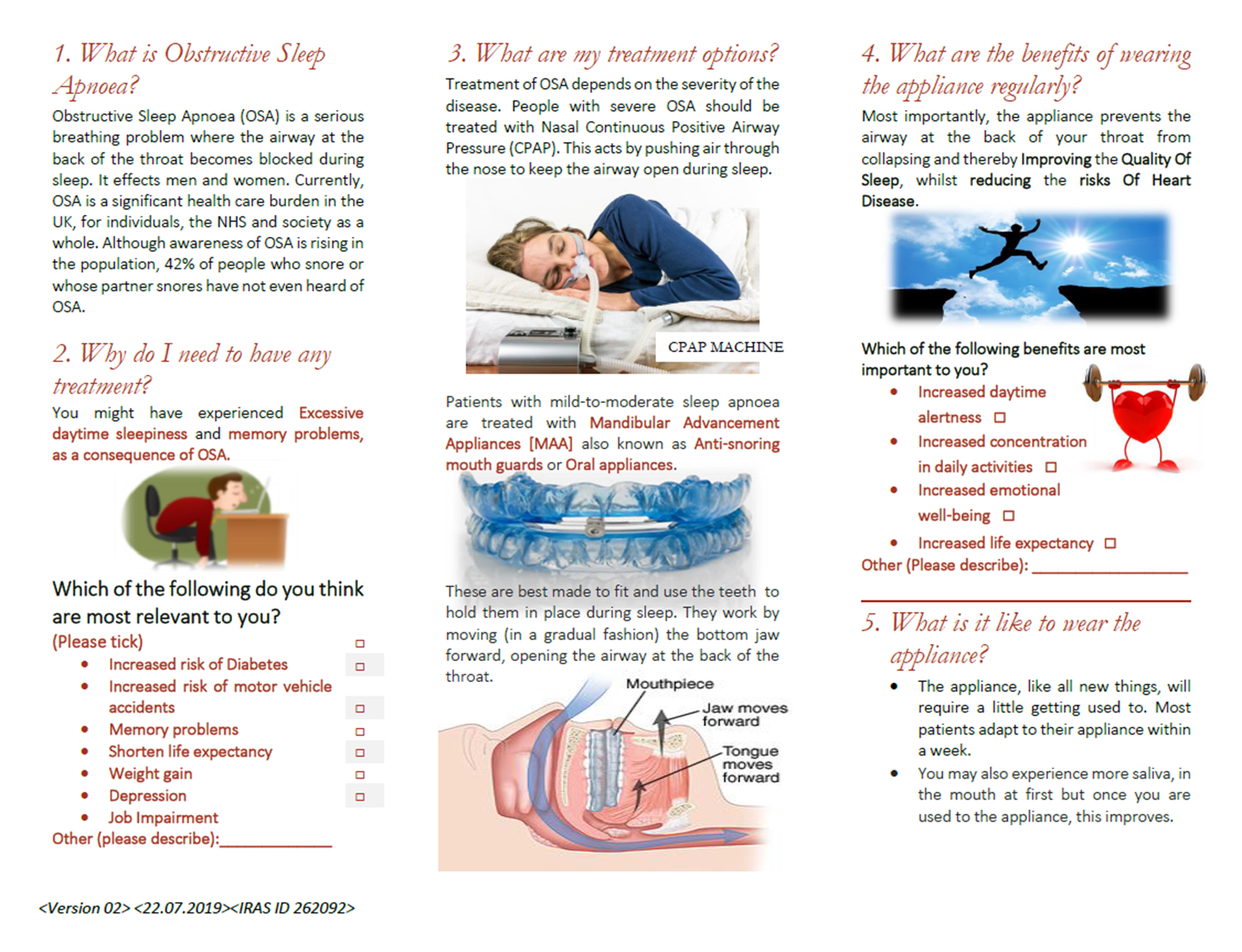


Appendix 2


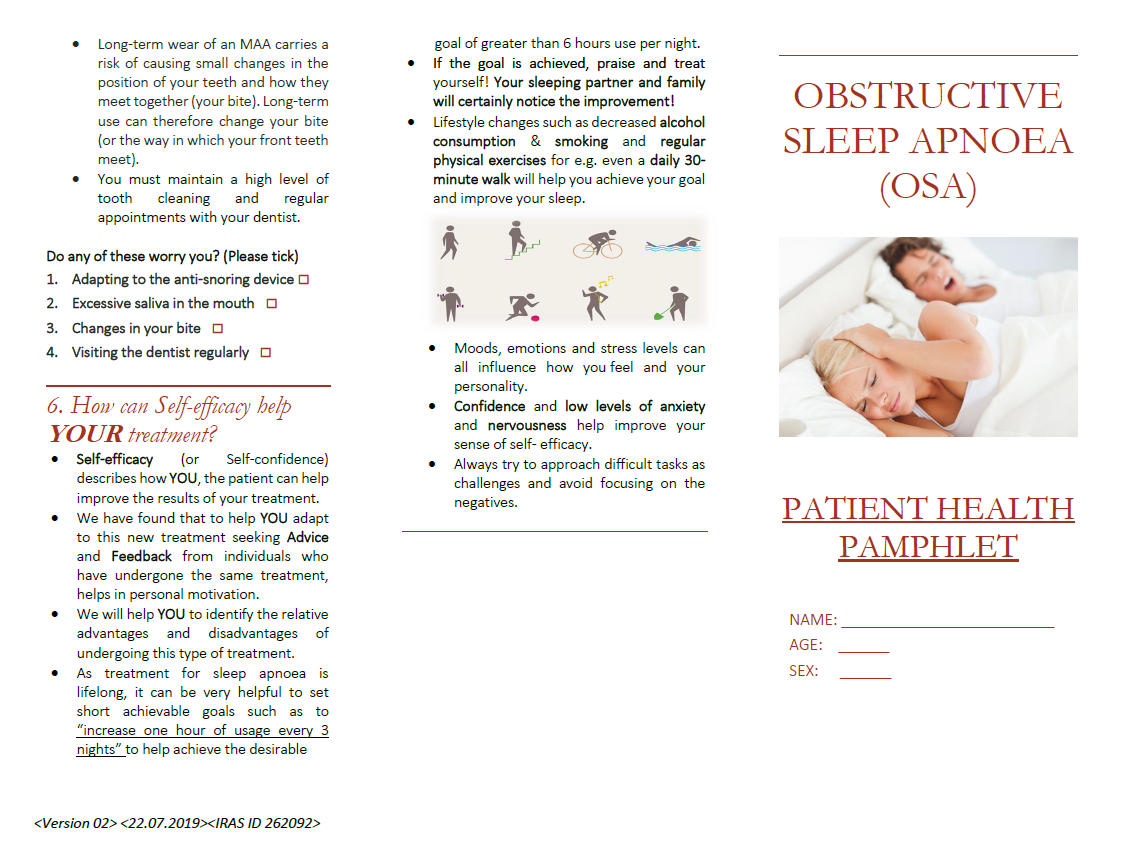

Supplement: Supplementary file 1 — Additional file 1. [file 13063_2021_5582_MOESM1_ESM.docx]
